# Yi Shen Juan Bi Pill Ameliorates Bone Loss and Destruction Induced by Arthritis Through Modulating the Balance of Cytokines Released by Different Subpopulations of T Cells

**DOI:** 10.3389/fphar.2018.00262

**Published:** 2018-03-27

**Authors:** Hongyan Zhao, Huihui Xu, Zhengyun Zuo, Gui Wang, Meijie Liu, Minghui Guo, Cheng Xiao

**Affiliations:** ^1^Experimental Research Center, China Academy of Chinese Medical Science, Beijing, China; ^2^Institute of Basic Theory for Chinese Medicine, China Academy of Chinese Medical Science, Beijing, China; ^3^Jiangxi University of Traditional Chinese Medicine, Nanchang, China; ^4^Institute of Clinical Medicine, China-Japan Friendship Hospital, Beijing, China; ^5^Beijing University of Chinese Medicine, Beijing, China

**Keywords:** Yi Shen Juan Bi Pill, bone destruction, bone loss, Th1, Th17, Tregs, collagen-induced arthritis

## Abstract

The Yi Shen Juan Bi Pill (YSJB), a traditional Chinese compound herbal drug, has been used as an anti-rheumatic drug in clinical practice. Cartilage and bone destruction of inflamed joints is the hallmark of rheumatoid arthritis (RA). Our previous study suggested that YSJB had a protective effect on joint damage in collagen-induced (CIA) rats. However, the role and the mechanism of YSJB in inflammation-induced bone loss are unavailable. The current study aimed to further evaluate the effect of YSJB on the joint destruction and the systemic bone loss, and to clarify the potential mechanism. CIA model was generated by using collagen II and incomplete Freund's adjuvant in Sprague-Dawley rats. After 4 weeks treatment, arthritic index, tissue pathology, micro-computed tomography scanning (μ-CT), and bone mineral density (BMD) analysis were performed. YSJB decreased arthritic scores and bone destruction; improved the BMD of lumbar vertebrae and bone volume fraction of inflamed joints. Moreover, YSJB significantly decreased the production of serum bone resorption markers, including Tartrate-Resistant Acid Phosphatase (TRACP), N-terminal telopeptide of type I collagen and C-terminal telopeptide of type I collagen. Meanwhile, it increased the level of serum bone formation marker type I collagen N-terminal propeptide. These results revealed that YSJB ameliorated bone destruction and reduced bone loss induced by arthritis. We have previously showed that Tregs inhibited osteoclast differentiation and bone resorption *in vitro*. Furthermore, others suggested that abnormality of Th1, Th17 may contribute to bone destruction. Here, we showed YSJB significantly up-regulated the percentage of Tregs, while also down-regulated the percentage of Th1 and Th17 cells. Our findings provide the evidence that YSJB ameliorates the severity of disease and joint degradation, and reduces systemic bone loss induced by arthritis. We propose YSJB modulates the balance of T cell phenotype, which affects the activation and differentiation of osteoclasts.

## Introduction

Yi Shen Juan Bi Pill (YSJB), which contains 20 medicinal materials, was developed by Professor Liangchun Zhu, a grand master of Chinese medicine science. YSJB has been extensively used for many years in local clinics of China for the treatment of rheumatoid arthritis (RA). RA is a common autoimmune disease characterized by chronic synovial inflammation and subsequent destruction of cartilage or bone. Focal bone destruction of inflamed joints is the hallmark of RA patients, resulting in functional disability and secondary osteoporosis (Koyama and Tanaka, 2016). Clinical trials suggested that YSJB ameliorated symptoms of RA patients and decreased rheumatic factor (RF), erythrocyte sedimentation rate (ESR), and C-reactive protein (CRP) (Li et al., 2008; Zhang et al., 2011). Additionally, our previous study demonstrated that YSJB had a protective effect on joint damage in collagen-induced (CIA) rats (Xiao et al., 2014). However, we know little about the role and mechanism of YSJB in regulating bone loss and bone destruction induced by arthritis.

It has been identified T helper (Th) cell subsets Th1/Th17, regulatory T cells (Tregs) and related cytokines are involved in RA (Gol-Ara et al., 2012; Wang et al., 2013; Wang H. et al., 2016; Wang T. et al., 2016). Activated Th1 and Th17 cells are found in RA synovial cavity in the process of inflammation (Park et al., 2017), and local abnormalities of Th1 and Th17 cells contribute to bone destruction of RA (Sato et al., 2006; Takayanagi, 2007; Adamopoulos and Bowman, 2008; Zhao E. et al., 2012). We have previously reported that YSJB significantly decreased IL-6, while increased IL-10 in serum of arthritis rats (Zhao H. et al., 2012). Excessive and prolonged activation of T lymphocytes, and together with mediators such as IL-6 and interferon gamma (IFNγ) play a vital role in the immunopathological processes in RA (Li et al., 2017).

Bone destruction caused by mature osteoclast is a frequent event in RA patients. Th17 regulates osteoclast differentiation through modulating cell to cell contact with osteoclast precursors (Behfarnia et al., 2013; Kikuta et al., 2013). Tregs play a vital role in the suppression of systemic bone destruction and limiting autoimmune inflammatory responses (Son et al., 2014). Based on our previous study showing an inhibitory effect of Tregs on osteoclast differentiation and bone resorption *in vitro* (Xu et al., 2016), we further hypothesized that the protective effects of YSJB on bone destruction and bone loss may occur through the regulation of Th1, Th17, and Treg cells in CIA rats.

## Materials and methods

### Animals

A total of 40 male SD rats (180–210 g) of 6–8 weeks were purchased from the National Institutes for Food and Drug Control [Animal license number: SCXK (Beijing) 2014-0013]. Rats were randomly divided into 4 groups with 10 in each group: control group, CIA group, YSJB group and Leflunomide (LEF) group. Rats were maintained with constant temperature of 22°C (±1°C) on a standard light/dark cycle (light phase from 6:00 to 18:00) environment with free access to standard rodent chow and water. All experimental procedures were examined and approved by the Institute of Basic Theory of Traditional Chinese Medicine, China Academy of Chinese Medical Sciences, Beijing, China (No: 2016028).

### Materials and chemicals

YSJB was purchased from Nantong Liangchun Hospital of Traditional Chinese Medicine (Nantong, Jiangsu, China). Leflunomide was manufactured by Clinkate Corporation (Suzhou, Jiangsu, China).

### Induction of CIA and evaluation of arthritis

Arthritis was induced as previously reported (Xiao et al., 2009). The rats were intradermally injected at the base of the tail with 100 μg of bovine type II collagen (Chondrex Inc., Redmond, WA, USA) emulsified in an equal volume of incomplete Freund's adjuvant (Chondrex). Rats were given a booster with the same preparation for 7 days after the primary immunization. On the 8th day after immunization, the degree of arthritis was examined every 3 days. The severity of arthritis was represented as arthritic index (AI) according to the following criteria: 0—no edema; 1—detectable edema and erythema limited to digits; 2—slight edema and erythema from digits to metatarsal bone; 3—moderate edema and erythema from foot to ankle; 4—maximal edema and erythema at entire ankle with ankylosis. The maximum AI scores per rat were 8 (4 points × 2 hind paws).

### Treatment of CIA

YSJB and LEF were orally administered 1.29 and 2.15 mg/kg·d to rats on the 15th day after primary immunization for 4 weeks. Rats in control group and CIA group were administered with an equal volume of pure water (1 ml/100 g).

### Histologic analysis

Blood was taken under anesthesia and rats were sacrificed by cervical dislocation 4 weeks after administration of drug. Both paws and ankle joints were dissected, fixed immediately for 3 days in formalin and the left were decalcified in 10% EDTA, and embedded in paraffin. Tissue sections were stained with hematoxylin and eosin (HE). Inflammation, pannus, cartilage damage, and bone damage were scored on a scale of 0–3 (0: absent; 1: weak; 2: moderate; 3: severe) (Zhao E. et al., 2012).

### Tartrate resistant acid phosphatase (TRACP) staining

The sections of ankle joints were subjected to TRACP staining (Sigma, St. Louis, MO, USA) to identify osteoclasts. TRACP^+^ multinucleated cells that containing 3 or more nuclei were identified as osteoclast and were counted. Specimens were evaluated by computer image analysis using the Leica Qwin image analysis software (Leica Microsystem, Germany).

### Micro-computed tomography (micro-CT) analysis

The right paws and ankle joints were scanned and reconstructed into a three-dimensional (3-D) structure by the SKYSCAN 1174 micro-CT (Bruker, Belgium). Bone volume (BV) and bone surfaces (BS) of the tarsal bones were then computed to evaluate the structural changes. To evaluate the surface density of the periarticular bone (indicating focal erosion on the bone surfaces) the ratio BS/BV was used (Marenzana et al., 2013).

### Bone mineral density (BMD)

After administration of drug, the lumbar vertebrae of all groups were removed and stored in −20°C. The lumbar vertebrae(L_4−6_)were measured by Osteocore3 Digital 2D (MEDILINK, France) to evaluate the BMD.

### Enzyme-linked immunosorbent assay (ELISA)

The procedures were performed according to the manufacturer's instructions. The serum levels of bone resorption markers, TRACP, N-terminal telopeptide of type Icollagen (NTX), C-terminal telopeptide of type I collagen (CTX), and bone formation markers osteocalcin (BGP), bone-source alkaline phosphatase (BALP), type I collagen N-terminal propeptide (PINP) were performed by Beijing Deyi Clinical Inspection Institute Co., Ltd, China. The level of IL-17A, IFN-γ, IL-10, and TGF-β1 in the serum was assessed using commercially available ELISA kits (eBioscience, San Diego, CA, USA).

### Isolation of the popliteal lymph node lymphocytes (PLNLs)

PLNLs of 4 groups were harvested separately after rats were sacrificed. Then, they were mechanically dissociated into phosphate-buffered saline and were washed twice. After centrifugation, the cells were collected at a density of 1 × 10^7^ cell/ml in RPMI-1640 supplemented with 10% FBS.

### Flow cytometry

Th1/Th17 cells were evaluated according to the manufacturer's protocol. PLNLs were incubated with a cell stimulation cocktail (plus protein transport inhibitors) for 5–6 h before staining. Next, PLNLs were isolated by centrifugation and stained with anti-Rat CD4 FITC (eBioscience) at room temperature in dark for 15 min. After fixation and permeabilization, PLNLs were stained with anti-Rat IL-17A (eBioscience) and anti-Rat IFN-γ or isotype controls (eBioscience) for 20 min at room temperature in dark. Finally, PLNLs were suspended in flow cytometry staining buffer after washing once and were then analyzed by BD FACSCalibur.

Flow cytometry was applied to identify Treg cells. PLNLs (approximately 1 × 10^6^) were first incubated with anti-rat CD4-FITC and anti-rat CD25-APC (eBioscience) for 15 min in dark at room temperature to stain the surface markers. Following fixation and permeabilization for intracellular staining, PLNLs were washed twice and incubated with anti-rat Foxp3-PE (eBioscience) in dark for another 30 min at room temperature. Finally, the stained cells were suspended in flow cytometry staining buffer after washing once and were analyzed by BD FACS Calibur.

### Statistical analysis

All of the data were analyzed using the SPSS 20.0 program and were expressed as the means ± standard deviation (SD) using one-way ANOVA. *P*-values less than 0.05 were considered to be statistically significant.

## Results

### Protective effect of YSJB on arthritis progression

**Figure 1:**
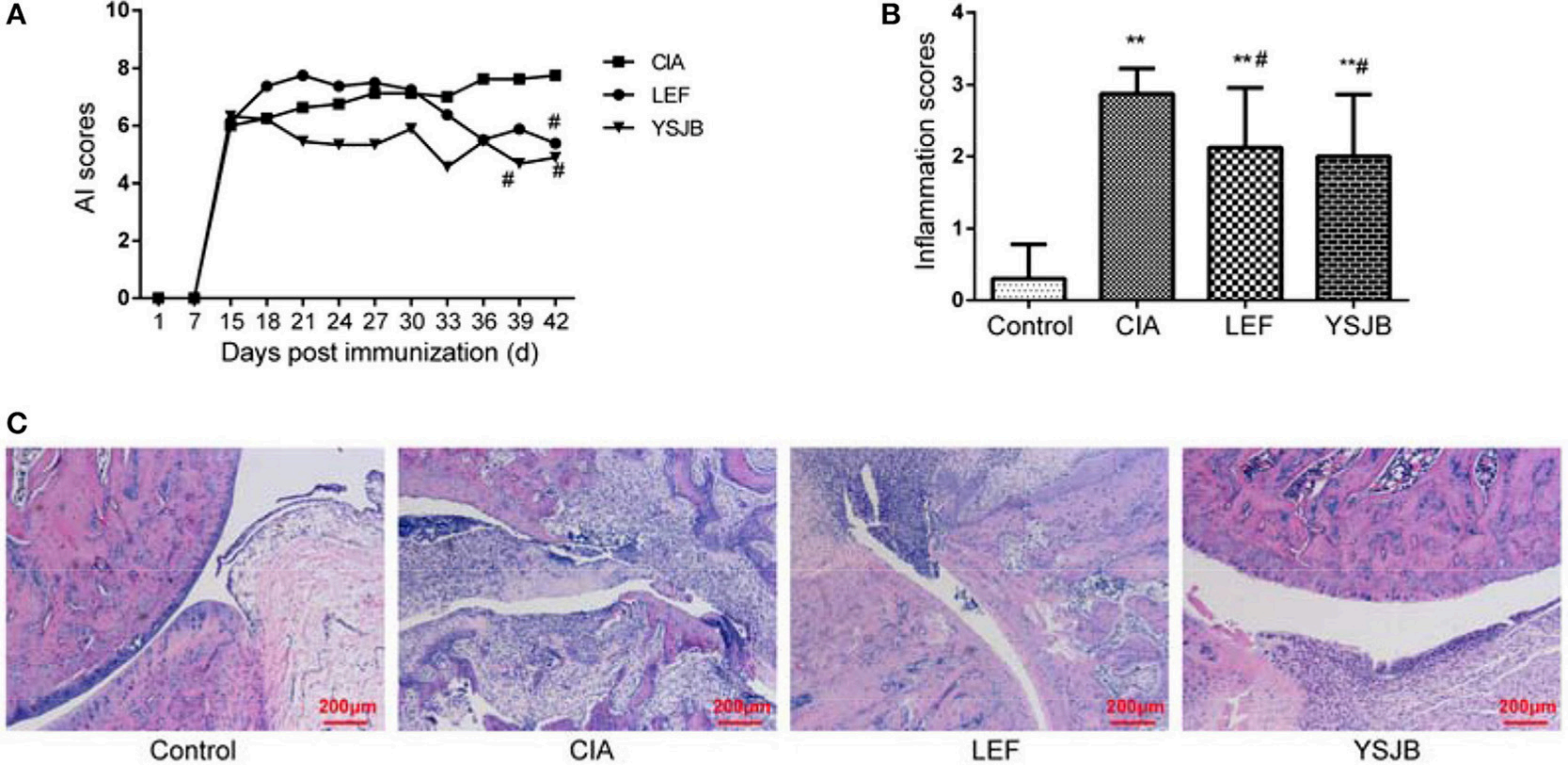
Effects of the YSJB on the progression of arthritis in CIA rats. Rats were orally administered with YSJB (1.29 g/kg·d), LEF (2.15 mg/kg·d), or pure water (both control group and CIA group) for 28 days on the 15th day after primary immunization. The arthritis and inflammation scores were evaluated. **(A)** Line plots represented the arthritis index of CIA group, LEF group and YSJB group. **(B)** Bar plots represented the inflammation scores of all the groups. **(C)** Representative pathological sections of the ankle joint by H&E staining. Data are presented as means ± *SD*. ^**^P < 0.01 compared with control group. ^#^*P* < 0.05 compared with CIA group.

Clinical signs of CIA first appeared in the paws and ankle joints on day 10 after immunization in Figure 1A. On day 18 after immunization, the AI scores started to decrease. YSJB lowered the arthritic scores compared with CIA group. Additionally, the arthritic scores in YSJB group and LEF group were significantly lower than those in CIA rats on days 39 and 42 (*P* < 0.05).

As shown in Figure 1C, histological evaluation of the ankle joints in CIA group showed inflammatory cell infiltration, synovial hyperplasia, cartilage and bone damage. Oral administration of YSJB and LEF could effectively reduce the extent of inflammatory cell infiltration and damage of cartilage and bone. In order to clarify the influence of YSJB treatment at the histological level, inflamed joints were scored with semiquantitative grading scales. As shown in Figure 1B, the inflammation scores after YSJB treatment were significantly decreased (*P* < 0.05).

### YSJB ameliorates bone destruction and bone loss in CIA rats

**Figure 2:**
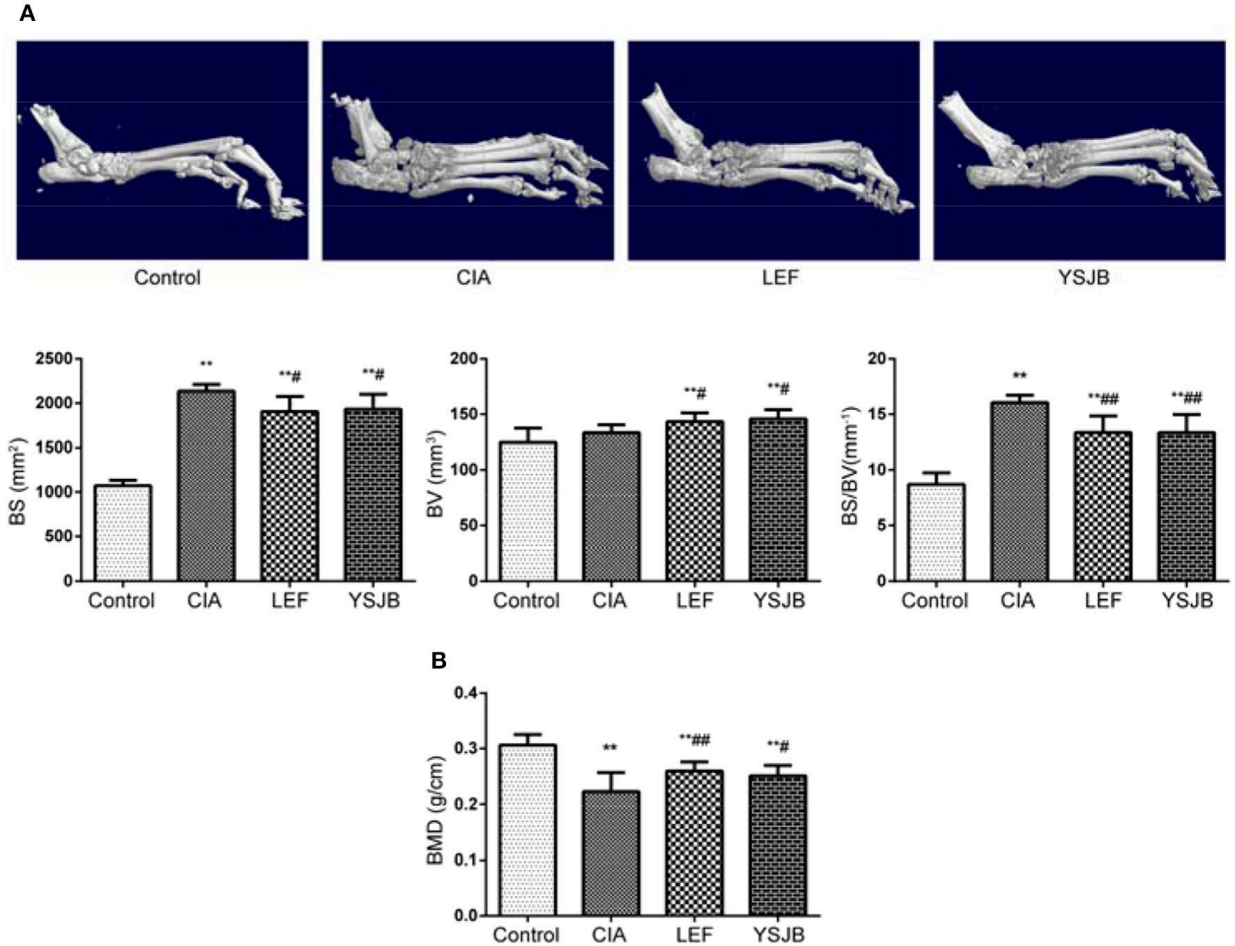
Micro-CT and dual energy X-ray scan demonstrate the bone-protective efficiency of YSJB on ankle joints and lumbar vertebrae in CIA rats. **(A)** Representative three-dimensional renditions of the ankle joint and bar plots of bone surface, bone volume, bone surface-to-bone volume ratio (BS/BV). **(B)** Bar plots represented BMD of the lumbar vertebrae. Data are presented as means ± *SD*. ^**^P < 0.01 compared with control group. ^#^*P* < 0.05, ^##^*P* < 0.01 compared with CIA group.

The 3-D picture of the hind paws and ankle joints in Figure 2A showed that the bone surfaces of CIA group was uneven, and the area of the tarsal surface increased obviously after analysis, but the BV had no obvious changes. After the treatment of YSJB and LEF, the surface area of the tarsal bone decreased (*P* < 0.05), bone mass increased (*P* < 0.05) and BS/BV decreased significantly (*P* < 0.01; Figure 2B). Moreover, we detected the BMD of the lumbar vertebrae by dual energy X-ray. Figure 2C shows that YSJB and LEF could effectively increase the BMD of lumbar vertebrae compared with CIA group (*P* < 0.05). These data suggested that YSJB and LEF could improve the bone destruction and increase the local and systemic bone mass caused by arthritis.

### YSJB inhibits osteoclast differentiation in inflamed joints

**Figure 3:**
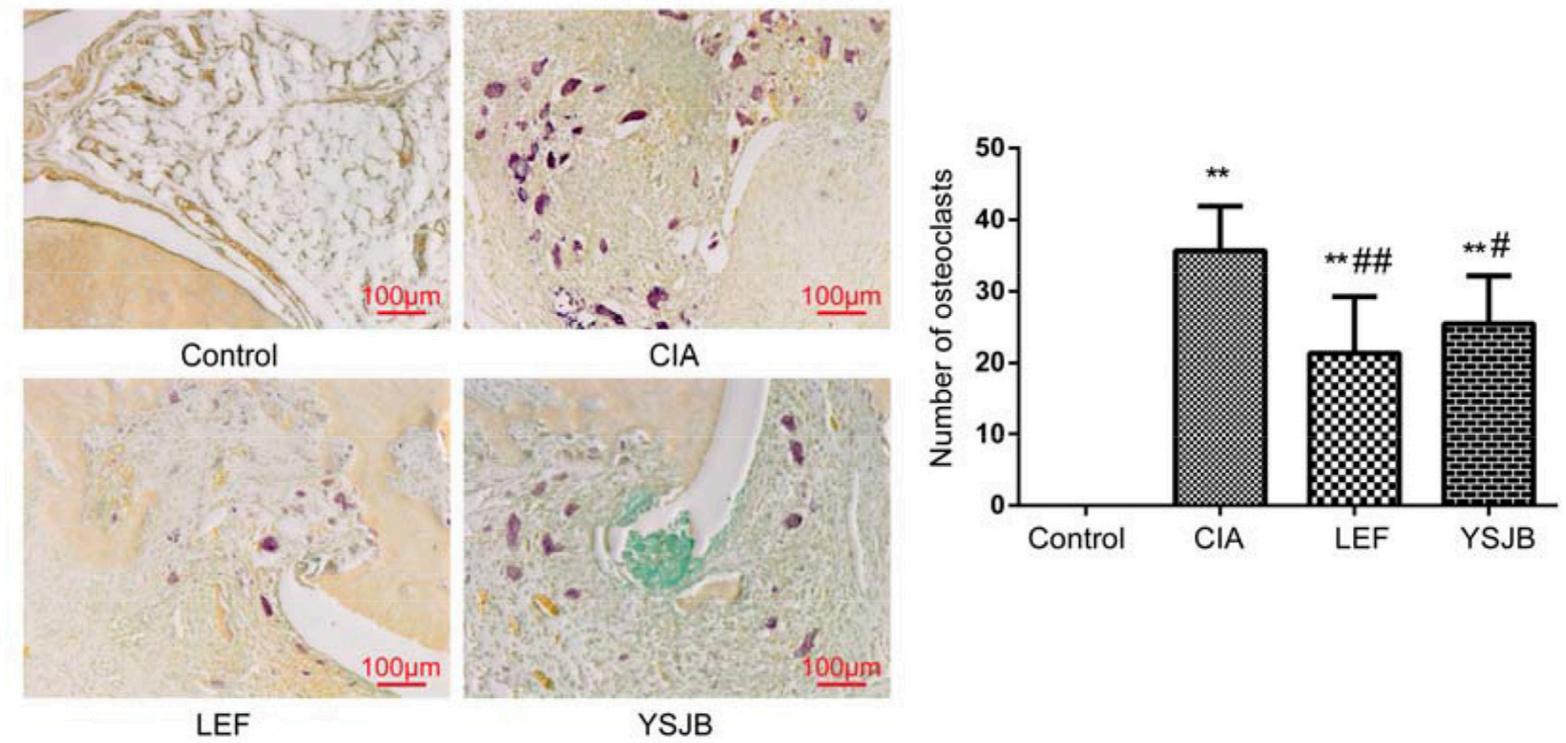
The YSJB inhibits osteoclast differentiation in CIA rats. The sections of ankle joints were stained with TRACP, and the number of osteoclasts was evaluated. The data are presented as means ± *SD*. ^**^P < 0.01 compared with control group. ^#^*P* < 0.05, ^##^*P* < 0.01 compared with CIA group.

Compared with CIA group, YSJB and LEF significantly reduced the number of osteoclasts in areas of bone destruction of the ankle joints (*P* < 0.05, *P* < 0.01, Figure 3). Thus, the inhibition of osteoclastogenesis is highly effective in suppressing bone destruction caused by arthritis.

### YSJB regulates the expression of serum markers on bone resorption and formation

**Figure 4:**
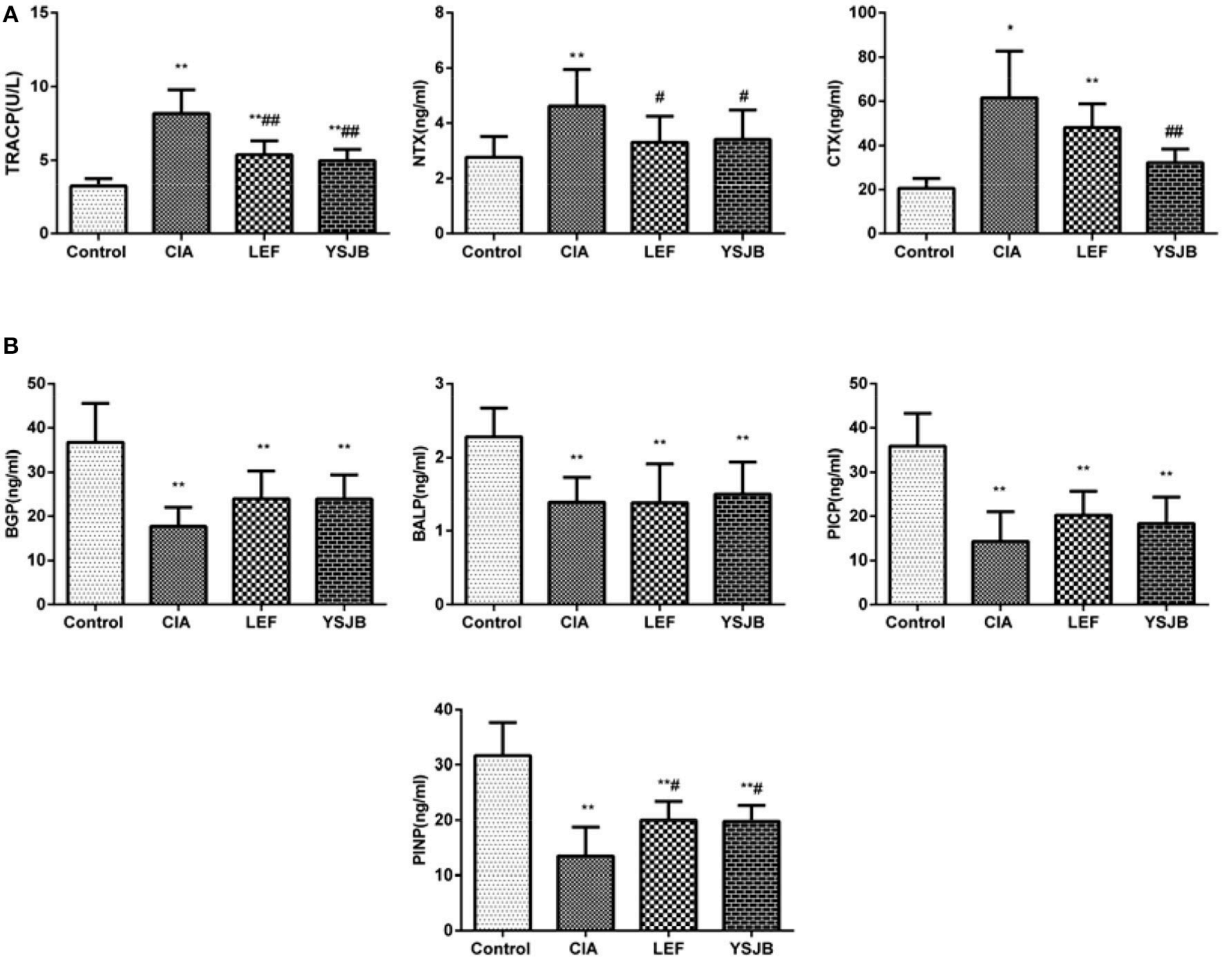
Regulation of bone markers after treated with YSJB in CIA rats. **(A)** Bar plots represented the markers of bone resorption with TRACP, NTX and CTX. **(B)** Bar plots represented the markers of bone formation with BGP, BALP PICP and PINP. The data are presented as means ± *SD*. ^*^P < 0.05, ^**^P < 0.01 compared with control group. ^#^*P* < 0.05, ^##^*P* < 0.01 compared with CIA group.

Markers of osteoclastogenesis and bone resorption, such as TRACP, NTX, and CTX, were significantly down-regulated in the YSJB group (*P* < 0.01, *P* < 0.05, Figure 4A). Meanwhile, the marker of bone formation, PINP, was up-regulated (*P* < 0.05) and BGP, BALP, PICP have a tendency without statistical difference (Figure 4B). This observation suggests that YSJB exerts a protective effect against bone destruction and bone loss mainly by inhibiting osteoclastogenesis.

### Regulation of Tregs, Th1, and Th17 cells by YSJB in CIA rats

**Figure 5:**
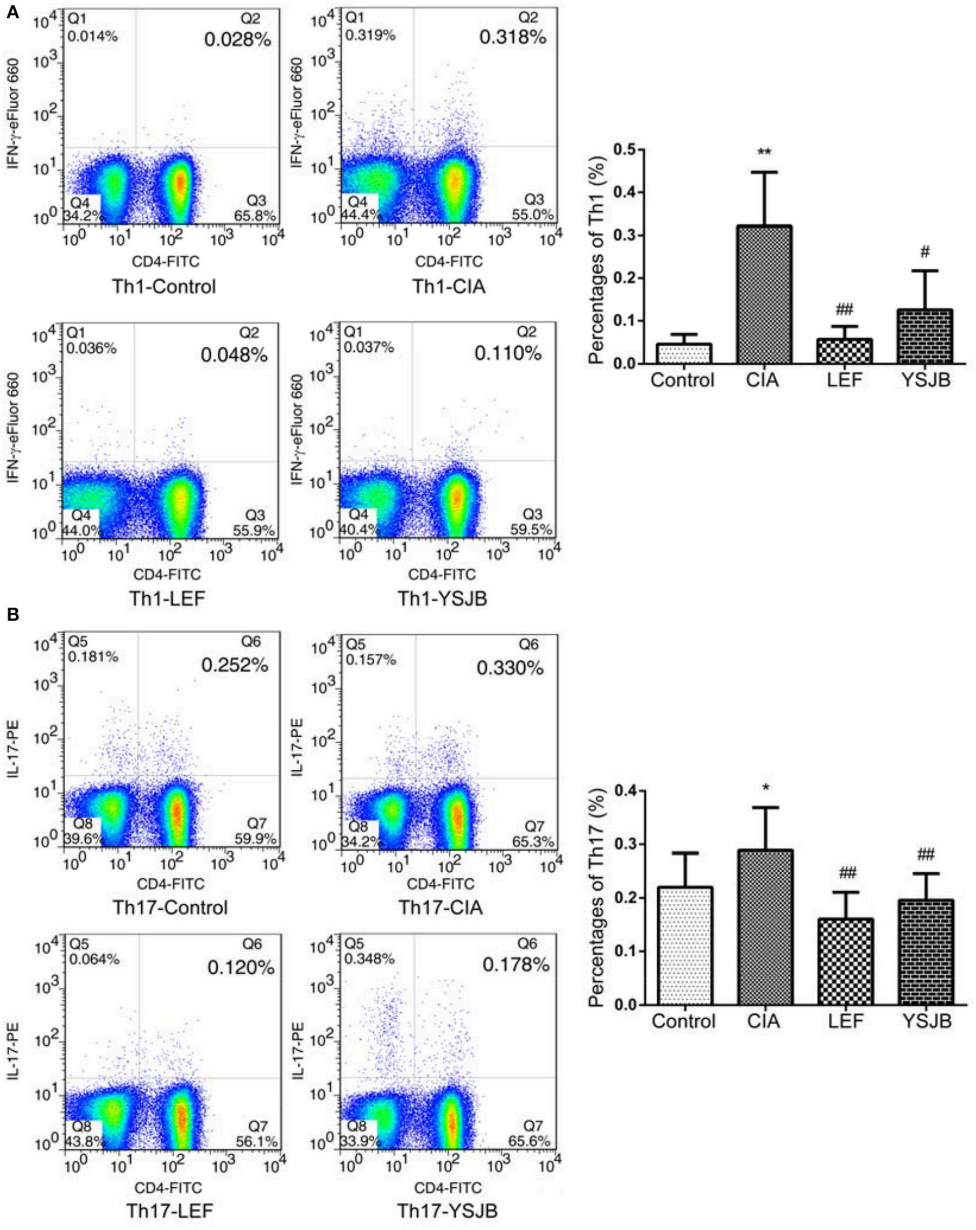
YSJB decreases the percentage of Th1 and Th17 cells obtained from the popliteal lymph node assessed by flow cytometry. **(A)** The percentage of Th1 cells are shown through staining with CD4, IFNγ. **(B)** The percentage of Th17 cells are shown through staining with CD4, IL-17. Data are presented as means ± *SD*. ^*^P < 0.05, ^**^P < 0.01 compared with control group. ^#^*P* < 0.05, ^##^*P* < 0.01 compared with CIA group.

**Figure 6:**
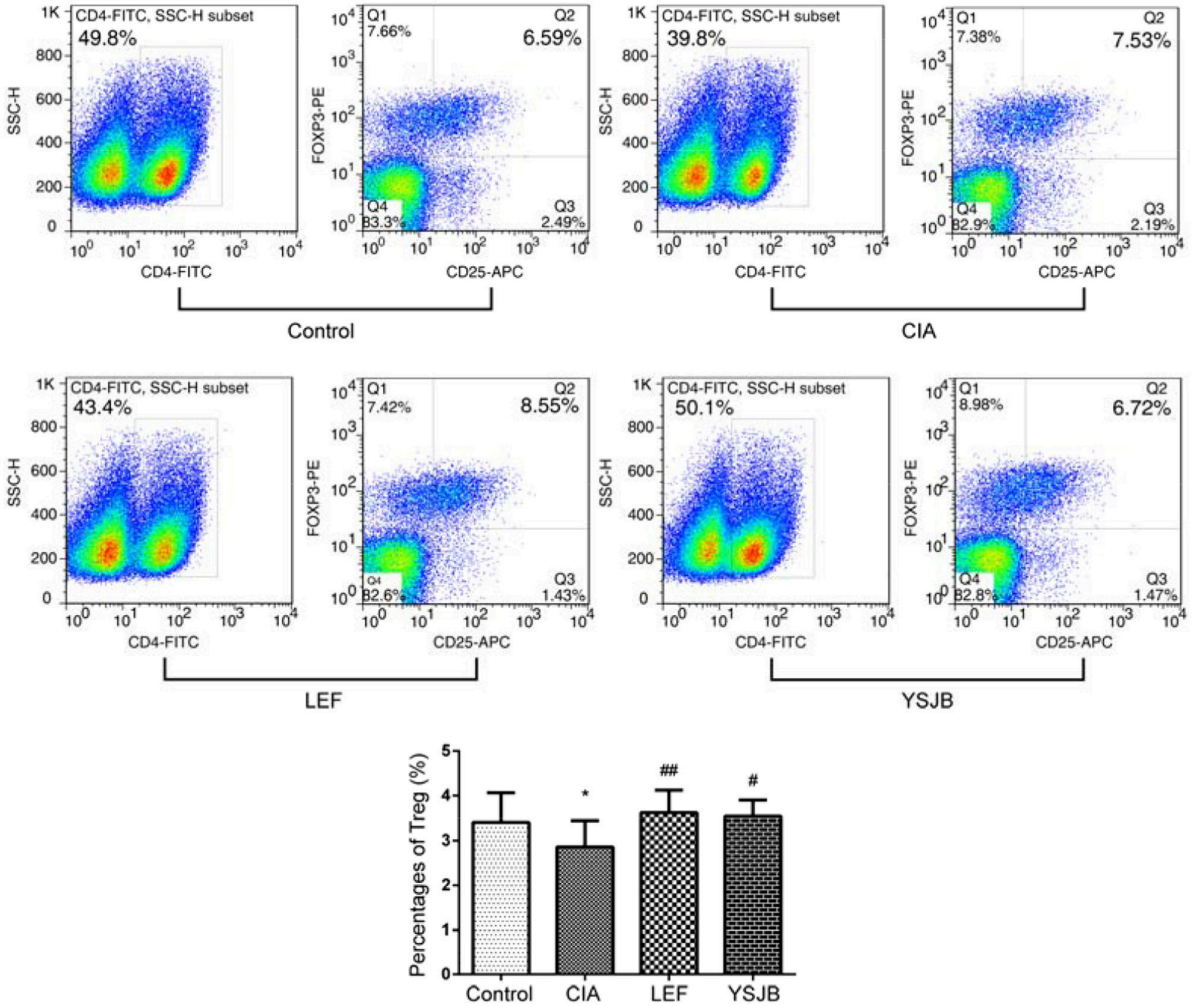
YSJB increases the percentage of Tregs obtained from the popliteal lymph node assessed by flow cytometry. The percentage of Tregs (CD4^+^CD25^+^FoxP3^+^) is shown through staining with representative antibodies against CD4, CD25, and FoxP3. The data are presented as means ± *SD*. ^*^P < 0.05 compared with control group. ^#^*P* < 0.05, ^##^*P* < 0.01 compared with CIA group.

T cells were obtained from the popliteal lymph node. The percentage of Tregs in the YSJB group and LEF group was significantly higher than that in CIA group (*P* < 0.01). By contrast, the percentage of Th1 and Th17 in the YSJB group was significantly lower compared to that in CIA group (*P* < 0.01, Figures 5, 6).

**Figure 7:**
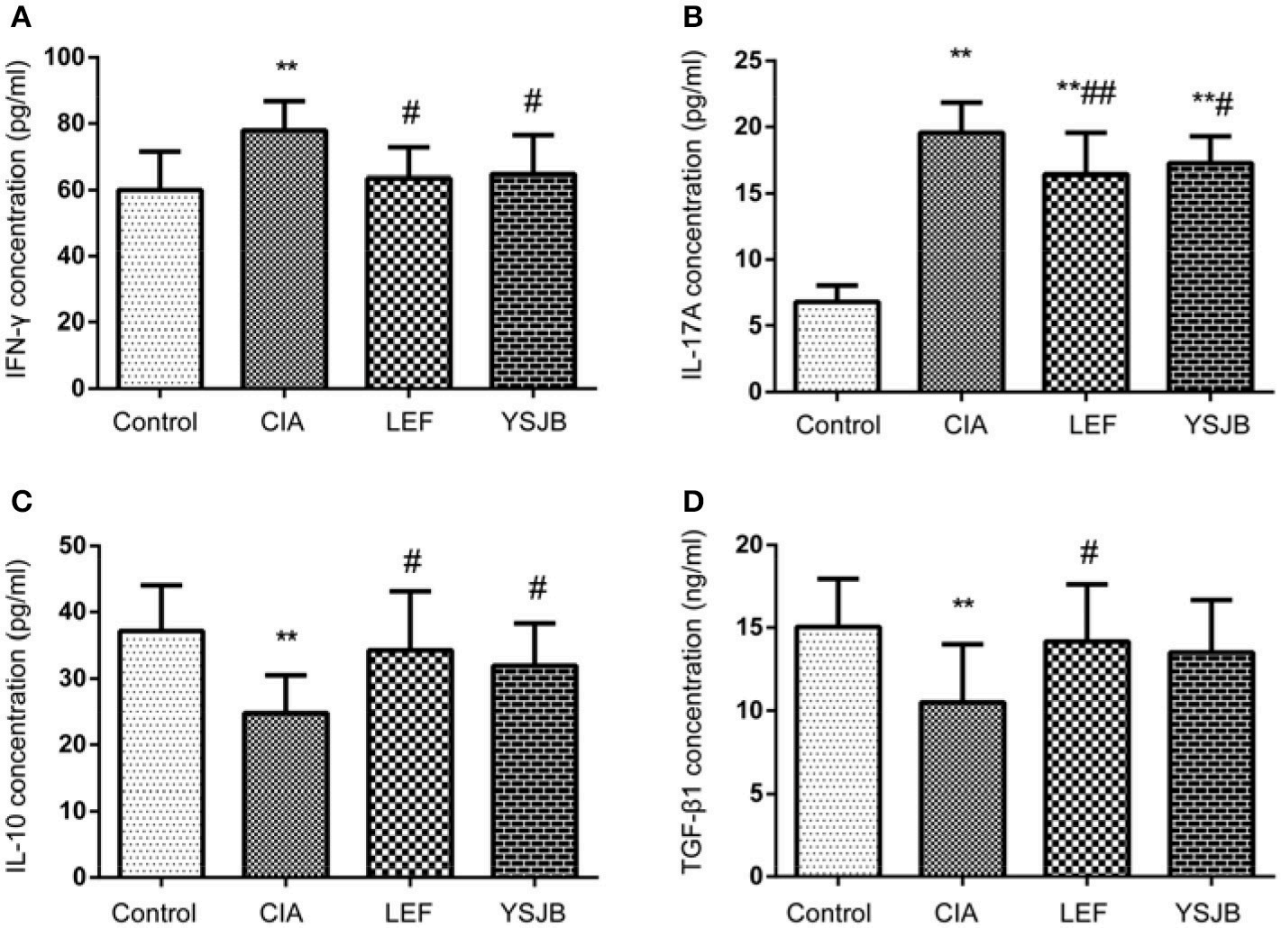
Regulation of cytokines treated with YSJB in CIA rats. Serum level was measured by ELISA. **(A)** Bar plots represented the mean levels of IFN-γ concentration of all groups. **(B)** Bar plots represented the mean levels of IL-17A concentration of all groups. **(C)** Bar plots represented the mean levels of IL-10 concentration of all groups. **(D)** Bar plots represented the mean levels of TGF-β1 concentration of all groups. The data are presented as means ± *SD*. ^**^P < 0.01 compared with control group. ^#^*P* < 0.05, ^##^*P* < 0.01 compared with CIA group.

Additionally, as shown in Figure 7, the level of both IFNγ and IL-17A in CIA group was higher, and the level of IL-10 and TGF-β1 was lower compared to those in control group (*P* < 0.01). YSJB significantly reduced the production of IFNγ and IL-17A and enhanced the level of IL-10 compared with that in CIA group (*P* < 0.05). TGF-β1 was also up-regulated without statistical difference. LEF also decreased the level of IFNγ and IL-17A (*P* < 0.05, *P* < 0.01), and increased the level of IL-10 and TGF-β1 compared with CIA group (*P* < 0.05).

## Discussion

RA is a chronic inflammatory autoimmune disease, driven by cellular, and humoral immune responses, which results in cartilage and bone destruction of inflamed joints (Mukai et al., 2015). Inflammatory bone destruction causes joint destruction and ultimately leads to functional disability. Clinically, three distinct bone alterations can be showed in RA: focal articular bone erosion, juxta-articular bone loss adjacent to synovitis, and systemic bone loss. However, secondary bone erosion and systemic osteoporosis caused by RA are the main cause of disability in RA patients. It is of great significance to prevent or even reverse the occurrence of secondary osteoporosis in RA to improve the prognosis of RA and life quality of patients. YSJB is a traditional Chinese herbal drug for RA therapy. As our previous study (Xiao et al., 2014), this research also showed that YSJB had significant therapeutic effect as assessed by AI scores (Figure 1A), inflammation scores (Figures 1B,C) and the area of bone surface (Figure 2A) in a rat model of RA. Moreover, we found that YSJB could ameliorate inflammation-induced focal bone erosion (Figure 2B) and systemic bone loss (Figure 2C).

Osteoclasts play the key role in the bone destruction and bone loss of RA. A balance between bone resorption mediated by osteoclasts and bone formation mediated by osteoblasts is required to maintain normal bone homeostasis (Hienz et al., 2015). If osteoclasts are overactive or abnormal, the balance of bone metabolism breaks and leads to bone destruction (Chen et al., 2016). In this study, the number of osteoclasts in the area of bone destruction of ankle joints in CIA rats significantly increased, concomitantly with increased serum level of bone resorption markers TRACP 5b NTX and CTX (Lin et al., 2016). In has been shown that high bone resorption is associated with increased urinary pyridinoline, deoxypyridinoline, urinary NTX and serum CTX (Gough et al., 1994; Garnero et al., 1999). YSJB significantly reduced the number of osteoclasts in the bone destruction areas of the ankle joints. We further confirmed that YSJB reduced the concentration of TRACP, NTX, and CTX significantly. These results demonstrated that YSJB increased the production of bone formation markers BALP, PINP, BGP, and PICP. Interestingly, only the concentration of PINP was significantly up-regulated in the YSJB and LEF groups. Therefore, YSJB has a greater effect in bone resorption than in bone formation.

Osteoclasts are derived from the monocyte macrophage lineage and are a potential therapeutic target against bone destruction and bone loss (Park and Ji, 2016). The immune system may influence the activation and differentiation of osteoclasts, especially in RA (Okamoto and Takayanagi, 2011). Several reports showed T cell subsets consisting of Th1, Th17, and Treg cells involved in the pathogenesis of RA (Karri and Sheela, 2017; Wang et al., 2017; Yang et al., 2017). Additionally, these T cell subsets play a crucial role in bone homeostasis (Son et al., 2014). The local abnormality of Th1, Th17, and Treg cells in the bone marrow of RA patients may partially contribute to bone destruction in the skeletal system (Wang T. et al., 2016). Th1 and Th17 cells promote bone loss partly through the up-regulated production of proinflammatory mediators (Park et al., 2017). The proinflammatory cytokine IL-17, especially IL-17A, as the primary cytokine of Th17 cells, exerts destructive activities in the joint (Leipe et al., 2014). Tregs, which are characterized by expressing Foxp3 in the nuclei, play a critical role in limiting autoimmune responses through regulating the adaptive and the innate immune systems. Tregs suppress osteoclast differentiation and bone resorption in a cytokine-dependent manner such as IL-10 and TGF-β1 (Luo et al., 2011). Th17/Th1 cells and Tregs are functionally antagonistic toward each other, and the decrease in Tregs and increase in Th17/Th1 cells are closely associated with CIA.

In this study, elevated IL-17A serum level in CIA group was associated with the development of bone destruction. YSJB or LEF significantly diminished the percentage of Th1 and Th17 cells compared with that in CIA group. The function of IFNγ produced by Th1 also contributes to bone loss. Our results support that YSJB, as well as LEF, could interfere with the expression of IFNγ, which may participate to slow down the development of bone destruction. The percentage of Tregs in YSJB and LEF group was significantly higher than that in CIA group, and YSJB could enhance the level of IL-10 compared with that in CIA group.

LEF is a first-line treatment for RA. LEF functions to inhibit both the metabolism of lymphocytes and osteoclastogenesis to reduce bone destruction (Kobayashi et al., 2004; Urushibara et al., 2004). Therefore, LEF was used as the control drug to investigate the therapeutic efficiency of YSJB. The study showed that YSJB had a similar effect as LEF in RA treatment. However, LEF cannot improve the body weight caused by immunization, while YSJB significantly ameliorate the body weight and their activities.

## Conclusions

**Figure 8:**
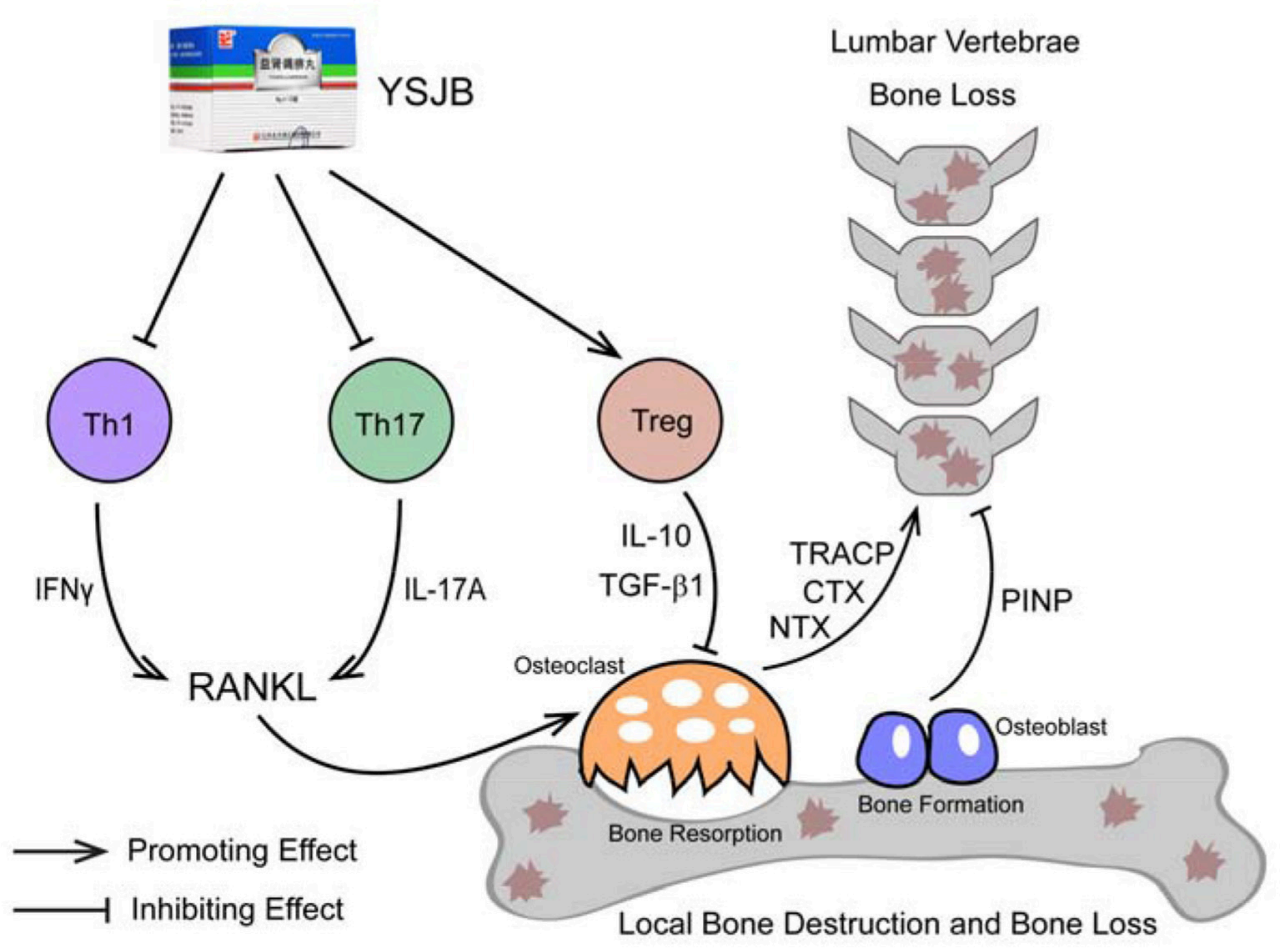
Mechanism of YSJB in treating CIA.

In summary, bone destruction and systemic bone loss is observed in a rat model of RA. The abnormality of Th1, Th17, and Treg cells in popliteal lymph nodes may partially contribute to bone destruction in the progress. The mechanism of YSJB ameliorating bone loss and destruction may be achieved by modulating the balance of T cell phenotypes to affect the activation and differentiation of osteoclasts (Figure 8).

## Ethics statement

This study was carried out in accordance with the recommendations of the Laboratory Animal Management Regulations, 1988 (China) (amended 2011), the Research Ethics Committee of Institute of Basic Theory for Chinese Medicine, China Academy of Chinese Medical Science. The protocol was approved by the Research Ethics Committee of Institute of Basic Theory for Chinese Medicine, China Academy of Chinese Medical Science.

## Author contributions

HZ, HX, and ZZ contributed equally to this paper. CX and HZ formulated the concept and designed the paper. HZ, GW, and HX performed the experiments. HZ and ML analyzed the data. HX and HZ drafted the paper. ZZ and MG participated in discussions related to the paper. ZZ, HZ, and CX revised the paper. All the authors read and approved the final paper.

### Conflict of interest statement

The authors declare that the research was conducted in the absence of any commercial or financial relationships that could be construed as a potential conflict of interest.
